# Factors affecting the mother’s choice of infant feeding method in Poland: a cross-sectional preliminary study in Poland

**DOI:** 10.1007/s11845-021-02751-8

**Published:** 2021-09-18

**Authors:** Anna Rozensztrauch, Magdalena Klaniewska, Marta Berghausen-Mazur

**Affiliations:** 1 Department of Pediatrics, Division of Neonatology, Wroclaw Medical University, Wroclaw, Poland; 2 Department of Pediatrics, Division of Pediatrics and Rare Disorders, Wroclaw Medical University, Wroclaw, Poland

**Keywords:** Breastfeeding knowledge, Breastfeeding support, Decision to breastfeed, Exclusive breastfeeding, Infant feeding method, Neonatal

## Abstract

**Background:**

The World Health Organization identifies exclusive breastfeeding as the gold standard for child nutrition. Breastfeeding provides many benefits to both the baby and the mother. First days after birth are crucial for breastfeeding and determining its further path. Unfortunately, very often it is also the time of facing the first breastfeeding problems. The aim of this study was to analyse selected factors influencing a mother’s decision to breastfeed.

**Methods:**

This is a cross-sectional study conducted between March 2019 and November 2019 in selected Polish maternity wards. Data were collected through completion of author’s questionnaire and a standardised questionnaire and the Multidimensional Perceived Social Support Scale and also through informal interviews with mothers. During statistical analysis, the chi-square test of independence and the Kolmogorov–Smirnov test as well as the non-parametric Mann–Whitney *U* test in addition to Fisher’s exact test were performed.

**Results:**

The results showed that there is no correlation between the mode of delivery and the mother’s choice of infant feeding method. Knowledge of the benefits of breastfeeding and received support affects the mother’s decision on infant feeding method. The study also showed that the 83% of newborns were put to the breast within the first 2 h after birth. However, only 58% continued to be breastfed in the subsequent days. As many as 42% of the newborns were supplemented with formula despite early initiation of breastfeeding. Analyses showed that exclusive breastfeeding was more often chosen by mothers with higher education. And the most common breastfeeding problem reported by 62% of the respondents was perceived insufficient milk supply and also breastfeeding pain, reported in 48.5% mothers.

**Conclusions:**

It is extremely important to continually promote breastfeeding among women. The role of midwives is crucial in this regard, as they should encourage the initiation of breastfeeding, support mothers during the perinatal and postnatal periods, and increase their sense of competence and confidence in their ability to breastfeed. Accessible, professional, and empathetic support can help reduce the number of women supplementing with modified milk. Undoubtedly, the postpartum period is important for breastfeeding outcomes, but it is significant that breastfeeding education and promotion begin during pregnancy.

## Background

Breast milk is widely acknowledged as the most complete form of nutrition for infants. It adapts to the changing needs of a newborn baby, whether full-term or premature. Exclusive breastfeeding is recommended by all child nutrition and health experts, including the World Health Organization (WHO); the European Society for Paediatric Gastroenterology, Hepatology and Nutrition (ESPGHAN); the United Nations International Children’s Emergency Fund (UNICEF); and the American Academy of Pediatrics (AAP) [1–3]. A global campaign ‘The Baby-Friendly Hospital Initiative’ was launched in 1989 by UNICEF and the WHO to advocate breastfeeding. Recent evidence indicates that breastfeeding could save over eight hundred thousand children’s lives and about two hundred mothers’ lives annually [4, 5].

Breastfeeding offers numerous benefits. It helps strengthen the bond between the mother and her newborn child; promotes the child’s physical, mental, and cognitive development; and reduces the risk of a number of conditions during the breastfeeding period and after weaning (i.e. obesity, diabetes, and hypertension). Breastfeeding boosts immunity, has a positive effect on the cardiovascular system, and supports the development of a healthy infant gut microbiome [6–8]. The protective properties of breastmilk are unique. All babies should be breastfed for at least the first 6 months of life [9]. Therefore, breastfeeding is considered the gold standard by child nutrition experts.

There is no doubt that the first days after birth are crucial for breastfeeding and determine its outcomes. It is then that the first contact between the mother and her newborn child, kangaroo mother care, and breastfeeding initiation take place. However, it is also the time when the first problems with breastfeeding occur.

Breastfeeding is to a large extent a matter of individual choice. Soon after birth, the mother needs to decide how to feed her newborn baby. It would seem that breastfeeding is so obvious and natural that it should be easy for every mother. However, a number of problems with breastfeeding often occur after birth. The psychological aspect is also important. It often takes a lot of perseverance and inner strength to breastfeed, especially one’s first child.

Even after making the decision to breastfeed, many mothers fail to reach their own breastfeeding goals because many factors discourage them from doing so. International studies have identified factors that affect breastfeeding intentions, such as public facilities for breastfeeding, social attitudes [10], employment [11], husband involvement [12], or personal perceptions [13].

Given the benefits of breastfeeding and the difficulties that breastfeeding mothers can experience, every effort must be made to support breastfeeding women, especially during the perinatal period. The role of midwives is crucial during that time. In the first days after birth, midwives are closest to mothers and their newborn children. As specialists in the promotion of breastfeeding, they have a strong influence on the mother’s decision whether to continue breastfeeding or not. Midwives are responsible not only for the provision of specialist assistance, but also for increasing young mothers’ sense of competence and confidence in their abilities e.g. to breastfeed.

This study was conducted to analyse selected factors influencing the mother’s decision to breastfeed. The specific objectives were to investigate (1) mode of delivery and its influence on the mother’s decision to breastfeed, (2) perceived support from family and its influence on the decision on infant feeding method, (3) knowledge of the benefits of breastfeeding and its influence on the breast feeding intention, (4) whether infants who are supplemented with formula at birth are less likely to be exclusively breastfed in the subsequent days, and (5) whether exclusive breastfeeding is chosen more often by mothers with higher education.

## Methods

### Design and participants

A cross-sectional survey was conducted between March 2019 and November 2019. The study included 103 female respondents. A total of 101 completed questionnaires were obtained; two were rejected as they had been filled out incorrectly. The inclusion criteria were as follows: provision of consent to participate in the study, correct completion of the questionnaires, at least 3 days postpartum, single uncomplicated pregnancy, mothers with no chronic diseases, full-term delivery, newborns born in a good general condition according to the Apgar score at 5 min (seven or more), and a birth weight of over 2000 g. The exclusion criteria were prematurity, mothers who were hospitalised due to postpartum complications, or mental illness.

### Data collection

Data were collected through completion of questionnaires and informal interviews. Information sheets outlining the purpose, significance, and design of the study were provided to the maternity unit concerned in order to clarify any issues and enlist their co-operation.

### Measures

The study was conducted using a diagnostic survey with the use of the authors’ own questionnaire as well as a standardised questionnaire, i.e. The Multidimensional Scale of Perceived Social Support, as adapted for use in Poland by Buszman and Przybyła-Basista [14–16]. Cronbach’s alpha for the questionnaire was 0.88.

The authors’ own questionnaire comprises 29 items, including questions regarding sociometric data (i.e. age, education, place of residence, marital status, and financial standing) and questions regarding breastfeeding (13 questions), newborn child (four questions), pregnancy (four questions), and birth (two questions). To analyse the tool’s reliability, its internal consistency was measured with Cronbach’s alpha. The value obtained was 0.963.

The Multidimensional Scale of Perceived Social Support includes 12 items scored on a 1–7 scale, where 1 denotes ‘very strongly disagree’ and 7 ‘very strongly agree’. The items concern the perception of support from family and friends. The questionnaire enables the assessment of total perceived social support (sum across all items), perceived support from a significant other (items 1, 2, 5, 10), perceived support from family (items 3, 4, 8, 11), and perceived support from friends (items 6, 7, 9, 12).

### Statistical analysis

During the statistical analysis, the chi-square test of independence was applied, which is a statistical hypothesis test used to determine whether there is an association between two categorical or nominal variables. The test makes it possible to determine whether a given idea is plausible or not. The Kolmogorov–Smirnov test was also applied. This is a non-parametric test for assessing the conformity of the distribution of the variables analysed to a normal distribution. This test is used for larger samples (*N* > 100 is given as one of the limits). It should be performed before starting any statistical analyses in order to select appropriate tests. It tests the null hypothesis, indicating a distribution close to a normal one. Values of *p* > 0.05 confirm that the assumption of a normal distribution is met. Moreover, the non-parametric Mann–Whitney *U* test was used. This is a non-parametric equivalent of Student’s *t*-test for independent samples used to compare two groups in terms of quantitative variables. It compares each observation with respect to the mean (not median) and ranks, and does not require the assumption of equality of the groups compared. Furthermore, Fisher’s exact test was used to determine if there were non-random associations between two categorical variables.

## Results

Sociodemographic details of the participants are shown in Table 1.

**Table 1:** Characteristics of the group studied

Age	Number (*N*)	Percentage (%)
Under 21	1	1.0
21–25	19	18.8
26–30	36	35.6
31–35	33	32.7
Over 35	12	11.9
	Number (*N*)	Percentage (%)
Marital status		
Single	19	18.8
Married	79	78.2
Divorced	3	3.0
Place of residence		
Rural area	35	34.7
Small town	22	21.8
Medium-sized town	9	8.9
Large town	35	34.7
Education		
Tertiary	62	61.4
Secondary	36	35.6
Primary	3	3.0
Financial situation		
Very good	27	26.7
Good	62	61.4
Moderate	12	11.9

### Verification of the study objectives

1. There is no correlation between the mode of delivery and the mother’s choice of infant feeding method.

To test this hypothesis, a chi-square test of independence was performed. The test result was not statistically significant (*p* > 0.05), which indicates that there is no correlation between the mode of delivery and the mother’s choice of infant feeding method (Table 2).

2. Perceived support affects the mother’s decision on infant feeding method.

In the first step of the analysis, the distribution of quantitative variables was tested. To this end, basic descriptive statistics were calculated and the normality of distribution was tested using the Kolmogorov–Smirnov test. The Kolmogorov–Smirnov test result was statistically significant for all the variables (*p* < 0.05). The results are presented in Table 3.

In addition, a comparative analysis of perceived support between breastfeeding women and women who exclusively formula-fed their babies was carried out. As the distribution of the variables was significantly different from normal distribution, the non-parametric Mann–Whitney *U* test was applied. The test results showed that there were no statistically significant differences (*p* > 0.05) between exclusively breastfeeding women and those who decided to mix-feed or exclusively formula-feed their babies both in terms of total perceived social support and in terms of particular dimensions of perceived social support. Detailed data are presented in Table 4.

3. Knowledge of the benefits of breastfeeding influences the mother’s feeding choices.

The results of the chi-square tests of independence showed that there is no statistically significant correlation (*p* > 0.05) between the choice of infant feeding method and the knowledge of such benefits of breastfeeding as faster uterine involution, stronger mother and child bond, and lower risk of diabetes.

In the next step, the association between the respondents’ knowledge of the benefits of breastfeeding and their choice of infant feeding method was analysed. To this end, the chi-square test and Fisher’s exact test were applied. In the case of knowledge about how breastfeeding strengthens the mother and child bond, Fisher’s exact test was used when the predicted count in any cell was less than 5.

The analysis showed a statistically significant relationship (*p* < 0.05) between the choice of infant feeding method and the knowledge that breastfeeding lowers the risk of ovarian cancer, and has a positive impact on a child’s intellectual development. However, the respondents who did not exclusively breastfeed had a higher awareness of those benefits. The women who used mixed feeding were more aware that breastfeeding lowers the risk of ovarian cancer compared to exclusively breastfeeding mothers (48.3% vs. 19.5%). Similar results were found for awareness of a positive impact of breastfeeding on a child’s intellectual development (55% of mix-feeding women vs. 14.6% of exclusively breastfeeding women). Despite being aware of the benefits of breastfeeding, the respondents supplemented breastfeeding with formula. Detailed data are presented in Table 5.

4. Infants who are supplemented with formula at birth are less likely to be exclusively breastfed.

In the next step, the correlation between the use of formula at birth and the decision to breastfeed in the subsequent days was analysed. Unfortunately, the majority (70%) of infants in the study were supplemented with formula. Only some respondents managed to establish exclusive breastfeeding in the days that followed (about 21%). The chi-square test of independence was used. The result of the test was statistically significant (*p* > 0.05), which means that there is a correlation between the use of formula at birth and the decision to exclusively breastfeed in the subsequent days. A higher proportion of respondents whose infants were not supplemented with formula at birth decided to exclusively breastfeed (Table 6).

5. Exclusive breastfeeding is chosen more often by mothers with higher education.

**Table 2:** Correlation between the mode of delivery and the choice of feeding method

Feeding method	Natural delivery		Natural delivery involving the use of oxytocin and/or vacuum extraction		Elective caesarean section		Emergency caesarean section	
	*N*	%	*N*	%	*N*	%	*N*	%
Exclusive breastfeeding	9	31.03	19	51.35	7	53.85	6	27.27
Mixed feeding or exclusive formula feeding	20	68.97	18	48.65	6	46.15	16	72.73
Total	29	100.00	37	100.00	13	100.00	22	100.00
*χ*^2^(3) = 5.44; ***p* = 0.142**								

A definition for the significance is *p* > 0.05

**Table 3:** Basic descriptive statistics for the variables analysed and the Kolmogorov–Smirnov test results

	*M*	Me	SD	Sk.	Kurt.	Min.	Max.	*D*	*p*
Support from a significant other	26.63	28.00	3.23	− 3.06	9.54	11.00	28.00	0.39	** < 0.001**
Support from family	25.83	28.00	3.59	− 2.40	6.41	11.00	28.00	0.27	** < 0.001**
Support from friends	24.91	27.00	4.21	− 1.55	2.02	11.00	28.00	0.25	** < 0.001**
Total perceived social support	77.39	81.00	10.20	− 2.50	7.02	34.00	84.00	0.26	** < 0.001**

*M* mean, *Me* median, *SD* standard deviation, *Sk.* skewness, *Kurt.* kurtosis, *Min*. *and Max.* minimum and maximum distribution value, *D* Kolmogorov–Smirnov test result; *p* significance of the Kolmogorov–Smirnov test

A definition for the significance is *p* > 0.05

**Table 4:** Comparative analysis of perceived social support between breastfeeding women and women who decided to use mixed feeding or exclusive formula feeding

	Exclusive breastfeeding (*n* = 41)				Mixed feeding or formula feeding (*n* = 60)				*Z*	*p*
	*M*	Me	Min	Max	Mean rank	Me	Min	Max		
Support from a significant other	52.16	28.00	14.00	28.00	50.21	28.00	11.00	28.00	− 0.42	0.677
Support from family	53.90	28.00	11.00	28.00	49.02	27.00	11.00	28.00	− 0.89	0.373
Support from friends	53.76	28.00	11.00	28.00	49.12	26.50	11.00	28.00	− 0.83	0.405
Total perceived social support	54.48	83.00	36.00	84.00	48.63	79.50	34.00	84.00	− 1.02	0.306

*n* number of observations, *M* mean, *Me* median, *Min. and Max.* minimum and maximum distribution value, *Z* Mann–Whitney *U* test result, *p* statistical significance of Mann–Whitney *U* test

**Table 5:** Benefits of breastfeeding

	Accelerates uterine involution					
	No		Yes			Test results
	*N*	%	*N*	%		
Feeding method						
Exclusive breastfeeding	12	29.27	29	70.73		*χ*^2^(1) = 0.37; *p* = 0.546
Mixed feeding or formula feeding	21	35.00	39	65.00		
Total	33	32.67	68	67.33		
	Lowers the risk of ovarian cancer					
	No		Yes			
	*N*	%	*N*	%		
Feeding method						
Exclusive breastfeeding	33	80.49	8	19.51		
Mixed feeding	31	51.67	29	48.33		
Total	64	63.37	37	36.63		
	Strengthens the mother and child bond					
	No		Yes			
	*N*	%	*N*	%		
Feeding method						
Exclusive breastfeeding	4	9.76	37	90.24		
Mixed feeding	6	10.00	54	90.00		
Total	10	9.90	91	90.10		
	Lowers the risk of diabetes					
	No		Yes			
	*N*	%	*N*	%		
Feeding method						
Exclusive breastfeeding	35	85.37	6	14.63		
Mixed feeding	43	71.67	17	28.33		
Total	78	77.23	23	22.77		
	Benefits a child’s intelligence and speech development					
	No		Yes			
	*N*	%	*N*	%		
Feeding method						
Exclusive breastfeeding	35	85.37%	6		14.63%	*χ*^2^(1) = 16.74; *p* < 0.001
Mixed feeding	27	45.00	33		55.00	
Total	62	61.39	39		38.61

**Table 6:** Correlation between the use of formula at birth and the mother’s choice on how to feed her baby in the subsequent days

	Was the baby given formula at birth?			
	Yes		No	
	*N*	%	*N*	%
Feeding method				
Exclusive breastfeeding	15	21.13	26	78.87
Mixed feeding or formula feeding	56	86.67	4	13.33
Total	71	100.00	30	100.00

The last objective analysed concerned the association between the education of the respondents and the choice of infant feeding method. In order to fulfil the chi-square test assumptions, women with primary education (three respondents) and women with secondary education (72 respondents) were grouped into one category.

The result of the chi-square test was statistically significant. This means that there is a correlation between the level of education of the respondents and the choice of infant feeding method. Exclusive breastfeeding was chosen more often by women with secondary and primary education compared to those with higher education (56.41% vs. 40.22%). Detailed data are presented in Table 7.

**Table 7:** Correlation between the level of education and the choice of infant feeding method

	Education			
	Higher		Secondary and primary	
	*N*	%	*N*	%
Feeding method				
Exclusive breastfeeding	19	40.22	22	56.41
Mixed feeding or exclusive formula feeding	43	59.78	17	43.59
Total	62	100.00	39	100.00
*χ*^2^(1) = 6.59; ***p* = 0.010**				

A definition for the significance is *p* > 0.05

### Analysis of the impact of early initiation of breastfeeding on breastfeeding success

The study showed that the vast majority of newborns (83%) were put to the breast within the first 2 h after birth. Over half of the newborns (58%) who were breastfed at birth continued to be breastfed in the subsequent days. As many as 42% of the newborns were supplemented with formula despite early initiation of breastfeeding. Seventeen babies (17%) were not put to the breast within the first 2 h after birth. Only six of them (35%) were exclusively breastfed in the subsequent days.

As many as 65% of the newborns who were not put to the breast at birth were bottle fed. Early initiation of breastfeeding increased breastfeeding success by 23%.

### Assessment of the impact of breastfeeding support from medical staff on the choice of infant feeding method

The majority (73%) of the respondents received breastfeeding support from midwives. However, 24% of those respondents considered the support to be insufficient. Approximately 9% of the women did not receive any support, while 19% did not need help with breastfeeding. However, over half (66%) of those respondents who did not receive support with breastfeeding used formula or mixed feeding.

### Analysis of the association between self-assessed breastfeeding knowledge and the choice of infant feeding method

The study showed that the vast majority (80%) of those respondents who used exclusive breastfeeding rated their breastfeeding knowledge as very good or good. Importantly, all the respondents who rated their breastfeeding knowledge as rather poor supplemented with formula.

### Sources of the respondents’ knowledge of breastfeeding

The item concerning the sources of information about breastfeeding was a multiple-choice question. The majority of the respondents (about 63%) received information about breastfeeding from midwives. Almost half of the women (48%) found information about breastfeeding on the Internet. Around 30% of the respondents indicated childbirth preparation classes, a family member, and/or literature as their source of knowledge about breastfeeding. A very small proportion of the respondents (4%) received information about breastfeeding from their physician.

### Analysis of breastfeeding problems

The study showed that perceived insufficient milk supply was the most common breastfeeding problem reported by the respondents. It was indicated by as many as 60 out of 97 respondents, i.e. 62%. Another major problem was breastfeeding pain. Almost half of the respondents (48.5%; *n* = 47) experienced pain when breastfeeding. Some respondents also reported such breastfeeding problems as difficulties finding a comfortable position (25.8%; *n* = 25) and unlatchable nipples (24.7%; *n* = 24). Breast fullness was also mentioned as a breastfeeding problem (15.4%). Only 8% of the respondents did not experience any problems when breastfeeding their babies.

The study showed that most of the respondents (65%) did not experience any infant-related breastfeeding challenges. Some babies were not very active during feeding (22%). This might have been due to physiological jaundice, which was diagnosed in 19% of the newborns. Ten per cent of the babies were diagnosed with excessive weight loss and a few had tongue-tie (2%).

## Discussion

All mothers should be supported to initiate breastfeeding. It is a complex process, which often requires mothers to show a lot of patience and perseverance, and deal with a number of breastfeeding problems [17, 18]. A study by Bień et al. [19] on the opinions and attitudes of women towards breastfeeding showed that mothers find the initiation of breastfeeding to be a difficult experience. In turn, a study by Wagner et al. [20] demonstrated that a vast majority of women experience difficulties at the early stage of breastfeeding. Our analysis showed that only 8% of the respondents did not report any breastfeeding problems. The most common breastfeeding problems were nipple pain, difficulty latching the baby on to the breast and finding a comfortable breastfeeding position, and breast fullness. Moreover, 62% of the respondents reported insufficient milk supply as the cause of their breastfeeding difficulties. Perceived insufficient milk supply is the main reason why mothers decide to use formula. Over 60% of mothers who use mixed feeding do so because they believe their milk supply is insufficient to satisfy their baby’s needs. These results are consistent with the literature on the subject [21, 23]. In a study by Li et al. [23], over 50% of mothers stated that they stopped breastfeeding at 1 or 2 months postpartum because they did not think they had enough milk. Breastfeeding success depends not only on the quantity of breastmilk produced but also on a number of different factors. Undoubtedly, one of them is a mother’s mental attitude towards breastfeeding [24]. This is why it is so important to provide lactation education at maternity units as well as to reinforce new mothers’ sense of competence and reassure them that the relatively small amount of breastmilk produced in the first days after birth is sufficient to satisfy their baby’s needs. Therefore, it seems that Nnebe-Agumadu et al. [25] are right in claiming that midwives who show mothers that they trust them have confidence in their parental abilities and appreciate their efforts help them feel confident and competent.

The literature confirms [26] that a large proportion of children are supplemented with formula in the first days after birth, even though in most cases there are no clear medical indications for the use of formula. The present study analysed the impact of the use of formula at birth on mothers’ future feeding choices. The study showed that there is a correlation between supplementation with formula at birth and the method of feeding in the subsequent days. Only some mothers (21%) managed to stop supplementing with formula and establish exclusive breastfeeding. The decision to introduce formula feeding (whether exclusive or partial) should be made by the physician in agreement with the mother, as unnecessary use of supplemental formula feeds disrupts lactation and shortens the duration of breastfeeding. Żukowska-Rubik et al. [27] concluded that individuals recommending the use of supplemental formula feeds should be familiar with the rules for assessing lactation performance and know how to assess the baby’s sucking technique and its effectiveness and, where necessary, intervene to improve the effectiveness of breastfeeding. The amount of supplemental formula feeds should be determined depending on the situation and the baby’s weight gain. The use of supplemental formula feeds of 60–80 ml, regardless of circumstances, may lead to the cessation of breastfeeding.

The present analysis of the impact of parity on a mother’s choice of infant feeding method revealed that the proportion of primiparous mothers who decided to supplement with formula was 12% higher than the proportion of multiparous mothers who used supplemental formula feeds. However, this factor has no impact on the incidence of breastfeeding problems, which were reported by the majority of the respondents. This is consistent with the results obtained by Gebuza et al. [28].

Recent years have seen growing caesarean section rates [29]. There is ongoing research investigating the difference in breastfeeding performance between women after natural delivery and those after caesarean section. Several studies have found that women after caesarean section are more likely to experience problems during the early stage of lactation and have more difficulties putting their babies to the breast [30–32]. There are several reasons that might account for the low prevalence of timely initiation of breastfeeding among mothers that had a caesarean section. Mothers who had a C-section might need some time to recover from anaesthesia or may not be comfortable adapting to a breastfeeding position. However, the present study did not confirm that there is a statistically significant correlation between the mode of delivery and the choice of infant feeding method. Likewise, Prior et al. [33] found no association between any type of C-section delivery and exclusive breastfeeding up to 6 months.

Many authors stress the importance of support provided to women after birth by a skilled attendant, especially in relation to breastfeeding [34–36]. A study by Britton et al. [37] showed that professional support has an impact on both the initiation and duration of breastfeeding. Support from partners is also important for breastfeeding mothers [38]. The present study found that women receive support from the people closest to them, i.e. their families, friends, and partners. However, the analysis did not show an association between perceived support and the choice of infant feeding method.

The present study also analysed the impact of education on the choice of infant feeding method. It was found that there is a correlation between the mother’s education and her feeding choice. In the group studied, the proportion of exclusively breastfeeding women with primary or secondary education was 16% higher compared with exclusively breastfeeding women with higher education. Day et al. [39] indicated that there is a tendency for mothers with higher education to never breastfeed their infants.

Mothers’ breastfeeding knowledge and its impact on their feeding choices have been analysed in a number of studies [40, 41]. A study by Suarez-Cotelo et al. [42], investigating factors that influence newborn or infant feeding choices, found that mothers with a higher level of knowledge are more likely to choose breastfeeding. The present study showed that the majority of the respondents were aware of the benefits of breastfeeding. However, this knowledge was not associated with the decision to exclusively breastfeed. It was also surprising that more mixed feeding mothers than exclusively breastfeeding mothers were aware of such benefits of breastfeeding as reduced risk of ovarian cancer and positive impact on a child’s intellectual development. As regards self-assessed breastfeeding knowledge, the present analysis showed that 80% of exclusively breastfeeding mothers rated their knowledge as ‘very good’ or ‘good’. On the other hand, all the respondents who considered their breastfeeding knowledge to be rather poor or very poor were supplementing with formula. Therefore, it may be concluded that it is not the level of knowledge that influences a mother’s feeding choice but rather her self-confidence and belief in her ability to breastfeed.

## Conclusion

While breastfeeding rates are high, a continuing effort needs to be made to further promote breastfeeding among women. Midwives should get involved in encouraging the initiation of breastfeeding and supporting breastfeeding mothers during the perinatal and postnatal periods, as it contributes to the success of breastfeeding, which is beneficial for both the mother and her baby. Professional aid during the lactation period and psychological support as well as assistance in solving problems through correction of errors and motivation may help reduce the number of women who supplement with formula. It is also clear that such care should also be provided after hospital discharge. Without doubt, the postnatal period is important for breastfeeding outcomes. However, the present study found that the majority of the respondents made the decision on how to feed their babies even before giving birth. Therefore, it is also important to ensure that education and promotion of breastfeeding begin already during pregnancy.

### Limitations

The findings of this study have to be seen in light of some limitations. First, the purpose of its cross-sectional design was not to determine causal correlations between the outcomes of interest and independent facts. Secondly, the methodology of data collection through face-to-face interviews, instead of self-administered questionnaires, could have resulted in overestimation of the practices related to exclusive breastfeeding because the respondents may have reported more socially desirable behaviours.

## Data Availability

The datasets generated for this study are available on request to the corresponding author.

## Abbreviations

WHO: World Health Organization
ESPGHAN: European Society for Paediatric Gastroenterology, Hepatology and Nutrition
UNICEF: United Nations International Children’s Emergency Fund
AAP: American Academy of Pediatrics
